# Nuclear jasmonate and salicylate signaling and crosstalk in defense against pathogens

**DOI:** 10.3389/fpls.2013.00072

**Published:** 2013-04-05

**Authors:** Selena Gimenez-Ibanez, Roberto Solano

**Affiliations:** Plant Molecular Genetics Department, Centro Nacional de Biotecnología-Consejo Superior de Investigaciones CientíficasMadrid, Spain

**Keywords:** jasmonates, salicylic acid, plant hormones, toxins, effector, plant resistance, susceptibility

## Abstract

An extraordinary progress has been made over the last two decades on understanding the components and mechanisms governing plant innate immunity. After detection of a pathogen, effective plant resistance depends on the activation of a complex signaling network integrated by small signaling molecules and hormonal pathways, and the balance of these hormone systems determines resistance to particular pathogens. The discovery of new components of hormonal signaling pathways, including plant nuclear hormone receptors, is providing a picture of complex crosstalk and induced hormonal changes that modulate disease and resistance through several protein families that perceive hormones within the nucleus and lead to massive gene induction responses often achieved by de-repression. This review highlights recent advances in our understanding of positive and negative regulators of these hormones signaling pathways that are crucial regulatory targets of hormonal crosstalk in disease and defense. We focus on the most recent discoveries on the jasmonate and salicylate pathway components that explain their crosstalk with other hormonal pathways in the nucleus. We discuss how these components fine-tune defense responses to build a robust plant immune system against a great number of different microbes and, finally, we summarize recent discoveries on specific nuclear hormonal manipulation by microbes which exemplify the ingenious ways by which pathogens can take control over the plant’s hormone signaling network to promote disease.

## INTRODUCTION

In nature, plants live in complex environments in which they intimately interact with a broad range of microbial pathogens with different lifestyles and infection strategies. To defend themselves against all these different types of pathogens, plants have evolved sophisticated strategies to perceive their attacker and to translate this perception into an effective immune response. Two tiers of recognition by the innate immune system have been defined (Jones and Dangl, 2006). The first branch is triggered by the recognition of highly conserved microbe-associated molecular patterns (MAMPs) by host cell transmembrane proteins that function as pattern recognition receptors (PRRs), which in turn, activate MAMP-triggered immunity (MTI; Jones and Dangl, 2006). This activates sufficient defense to resist non-pathogenic microbes and probably also, some pathogens. To overcome such line of defenses, adapted pathogens have acquired the ability to introduce virulence effector proteins into the plant cell to promote plant susceptibility (Jones and Dangl, 2006). The second branch recognizes microbial effectors inside the plant cell via nucleotide-binding site-leucine-rich repeat (NB-LRR) resistance (R) proteins (Jones and Dangl, 2006). This leads to activation of effector-triggered immunity (ETI), and is characteristically associated with programmed cell death known as the hypersensitive response (HR; Jones and Dangl, 2006). The HR lesion is a stronger form of defense and limits microbial spread by killing infected plant cells. The final outcome of the battle depends on the balance between the ability of the pathogen to suppress the plant’s immune system and the capacity of the plant to recognize the pathogen and to activate effective defenses.

The regulation of the defense network that translates the pathogen-induced early signaling events into activation of effective defense responses depends profoundly on the action of plant phytohormones (Pieterse et al., 2012). These hormones are small signal molecules occurring in low concentrations, essential for the regulation of plant growth, development, reproduction and survival to stresses of biotic and abiotic origin (Robert-Seilaniantz et al., 2011). Upon pathogen attack, the quantity, composition and timing of the phytohormonal blend produced by the plant varies among plant species and depends greatly on the lifestyle and infection strategy of the invading attacker (De Vos et al., 2005). Classic phytohormones are abscisic acid (ABA), auxins, cytokinins (CKs), ethylene (ET), and gibberellins (GAs), but small signaling molecules such as brassinosteroids (BRs), jasmonates (JAs), and salicylic acid (SA) are recognized as phytohormones as well (Pieterse et al., 2012). The importance of JA and SA as primary signals in the regulation of the plant’s immune response is well established (Loake and Grant, 2007; Robert-Seilaniantz et al., 2011; Pieterse et al., 2012). The JA pathway is primarily induced by and effective in mediating resistance against herbivores and necrotrophic pathogens, whereas the SA pathway is primarily induced by and effective in mediating resistance against biotrophic pathogens (Glazebrook, 2005). JA and SA defense pathways generally antagonize each other and thus, elevated resistance against necrotrophs is often correlated with increased susceptibility to biotrophs, and *vice versa *(Grant and Lamb, 2006). This is, however, an overly simplistic view of the complex repertoire of plant hormones that probably play a role in mediating inducible defenses. Indeed, ABA, auxins, BRs, CKs, ET, GAs, and additional oxylipins (other than JA) function as modulators of the plant immune signaling network as well, fine-tuning the hormonal balances to become more resistant to the invading organism (Robert-Seilaniantz et al., 2011; Pieterse et al., 2012; Vicente et al., 2012). The collective contribution and timing of these hormones during plant–pathogen interactions is crucial to the success of the interaction.

Typically, hormone signaling pathways begin with perception of a ligand hormone by a receptor and continue with the propagation of the hormone signal, leading to massive changes in gene expression within the nucleus (Lumba et al., 2010). In some cases, the perception and propagation of the signal initiates in the cytoplasm and then translocates to the nucleus. This is the case for SA, ABA, CK, and ET (Santner and Estelle, 2009; Fu et al., 2012; Pieterse et al., 2012; Wu et al., 2012). However, several plant hormone receptors are located directly in the nucleus. This is the case for the JA, auxins, and GAs hormonal pathways (Fonseca et al., 2009a; Kelley and Estelle, 2012; Pieterse et al., 2012). Although plant nuclear receptors are not transcription factors (TFs) *per se*, as is the case for animal nuclear receptors, they act directly on or just upstream of transcriptional regulators (Chini et al., 2009a; Fonseca et al., 2009a; Lumba et al., 2010). This shortened pathway yields simple and direct control of gene expression that is directly responsive to ligand concentrations. This results in a fast activation of a specific set of defense-related genes that determines the nature and effectiveness of the immune response that is triggered by the attacker (De Vos et al., 2005).

Recent discoveries highlight the importance of the 26S ubiquitin-proteasome system (UPS) in phytohormone signaling (Kelley and Estelle, 2012). In fact, UPS-mediated protein degradation has been demonstrated for every plant hormone, including ABA, auxin, BR, CK, ET, GA, JA, and recently SA (Chini et al., 2009a; Kelley and Estelle, 2012). UPS regulates hormone biosynthesis, transport, and perception and thus provides a simple and direct mechanism to control hormone signaling by the selective destruction of proteins whose concentrations must vary with time and alterations in the state of the cell. Interestingly, most of the hormone-related targets for UPS degradation described to date in plants are nuclear proteins associated with transcriptional repression that contain an ETHYLENE RESPONSE FACTOR-associated amphiphilic repression (EAR) domain (Ohme-Takagi and Shinshi, 1995; Kelley and Estelle, 2012). For example, the auxin/indole-3-acetic acid (Aux/ IAA; auxin), some jasmonate-ZIM domain (JAZ; JA), and the BRASSINAZOLE RESISTANT1 (BZR1; BR) repressors (Kagale et al., 2010). Thus, nuclear protein turnover is an integral and critical component in hormone signaling to ensure a fast and appropriate level of defense responses to a specific pathogen.

Here, we review the most recent and outstanding examples regarding hormonal crosstalk at the molecular level, focusing on the jasmonate and salicylate pathways and how the newly identified nuclear components fine-tune defense responses to build a robust plant immune system against a great number of different microbes. We also describe some of the best characterized molecular examples of specific nuclear hormonal manipulation by microbes, which exemplify the ingenious ways by which pathogens can take control over the plant’s hormone signaling network to suppress host immunity.

## JASMONATE AND SALICYLATE: MAJOR PLAYERS IN PLANT IMMUNITY

Plant immunity strongly relies on two plant mutually antagonistic hormones, JA and SA (Glazebrook, 2005). Both hormones control defense responses to different types of microbes and thus, they orchestrate a different and complex transcriptional reprogramming that eventually leads to plant resistance. Receptors of both hormones as well as many components of their signaling pathways have been recently identified. These discoveries are facilitating the understanding of the role of these hormones in plant immunity.

### THE JASMONATE PATHWAY

Jasmonates are lipid-derived molecules originating from α-linolenic acid from the plastid membrane (Schaller and Stintzi, 2009). Among the plant hormones, JA plays a key role in modulating many physiological processes and is a key cellular signal involved in the activation of immune responses to most insect herbivores and necrotrophic microorganisms (Glazebrook, 2005; Wasternack, 2007). Among all JAs found in nature, (+)-7-iso-JA–L-Ile is the molecularly active form of the hormone (Fonseca et al., 2009b). JA-isoleucine (JA-Ile) is perceived through a co-receptor complex formed by the F-box protein CORONATINE-INSENSITIVE 1 (COI1) and JAZ proteins, a family that comprise 12 members in *Arabidopsis* (Chini et al., 2007, 2009b; Thines et al., 2007; Sheard et al., 2010). COI1 is a nuclear F-box component of an SCF-(Skip-cullin-F-box)-type E3 ubiquitin ligase required for all JA-dependent responses tested so far (Feys et al., 1994; Xie et al., 1998; Katsir et al., 2008; Chini et al., 2009a; Fonseca et al., 2009a). *Arabidopsis *plants lacking the* COI1 *gene are more susceptible to necrotrophic pathogens such as *Alternaria brassicicola* and *Botrytis cinerea* (Thomma et al., 1998; Lorenzo et al., 2003), whereas these plants are more resistant to biotrophic bacterial pathogens such as *Pseudomonas syringae*, and show elevated SA levels consistently with SA-JA antagonism (Kloek et al., 2001). JAZ co-receptors are COI1 substrates that negatively regulate the JA-signaling pathway by directly interacting with and repressing TFs that control JA-regulated genes (Chini et al., 2007, 2009b; Thines et al., 2007; Sheard et al., 2010; Fernandez-Calvo et al., 2011; Pauwels and Goossens, 2011). In basal conditions, repression of TFs by JAZ proteins require the recruitment of the co-repressor TOPLESS (TPL) and TPL-related proteins (TPR) by the adaptor protein NINJA (Pauwels et al., 2010). Upon elicitation, the hormone-triggered interaction of the COI1-JAZ co-receptor induces the ubiquitination and degradation of JAZ repressors liberating the TFs from NINJA and TPL and activating the transcriptional responses mediated by the hormone (Chini et al., 2007; Maor et al., 2007; Thines et al., 2007; Saracco et al., 2009; Pauwels et al., 2010; Sheard et al., 2010). Recently, a TF-dependent mechanism for nuclear import of cognate JAZs transcriptional repressor has been reported in *Arabidopsis*, supporting the general belief that the JA receptor complex functions within the nucleus (Withers et al., 2012). Complex formation in the presence of the hormone further requires inositol pentakisphosphate (InsP5) as a COI1 cofactor that potentiates the strength of the COI1–JAZ interaction (Sheard et al., 2010; Mosblech et al., 2011). This sustains previous observations showing that *Arabidopsis* mutants with altered levels of inositol polyphosphates displayed aberrant JA-dependent responses including altered defense capabilities to the insect *Plutella xylostella *(Mosblech et al., 2011).

Several TFs responsible for activation of different JA-mediated responses have been identified and include the basic helix-loop-helix (bHLH) TFs MYC2, MYC3, and MYC4 (Chini et al., 2007; Cheng et al., 2011; Fernandez-Calvo et al., 2011; Niu et al., 2011). Fernandez-Calvo et al. (2011) showed that MYC3 and MYC4 are activators of JA-regulated programs that act additively with MYC2 to regulate specifically different subsets of the JA-dependent transcriptional response. Interestingly, a triple mutant *myc2myc3myc4* in *Arabidopsis* is as impaired as *Arabidopsis* plants lacking the *COI1* gene in the activation of JA-dependent defense responses against insect herbivory by *Spodoptera littoralis* and the bacterial pathogen *Pseudomonas syringae *(Fernandez-Calvo et al., 2011), indicating that JA-dependent defense responses to these pathogens and pests are mostly controlled by these three TFs. Additional JAZ TF targets have been identified in the last 2 years. These include other bHLH TFs such as GL3 (GLABRA3), EGL3 (ENHANCER of GLABRA3) and TT8 (TRANSPARENT TESTA8), and the R2R3 MYB TFs PAP1 (PRODUCTION OF ANTHOCYANIN PIGMENT1), GL1, MYB75, MYB21, and MYB24 among others (Qi et al., 2011; Song et al., 2011). However, despite they are known to be involved in several physiological processes, the contribution of most of these TFs to resistant against pathogens remains unknown.

### THE SALICYLIC ACID PATHWAY

Salicylic acid is a secondary metabolite produced by a wide range of organisms (An and Mou, 2011). In plants, SA functions as a plant hormone required for innate immunity against biotrophic pathogens such as the bacteria *Pseudomonas syringae* (Vlot et al., 2009). Despite the key role of SA in immunity against microbial infections, how plants detect the hormone has remained unclear until very recently. Using different ligand/receptor-binding methods, two research groups reported that NPR1 (NON-EXPRESSOR OF PR GENES1) or NPR1-related proteins, NPR3 (NPR1-LIKE PROTEIN3) and NPR4 (NPR1-LIKE PROTEIN4), are the long-sought SA receptors in *Arabidopsis* (Fu et al., 2012; Wu et al., 2012). Wu et al. (2012) provided evidence, using a special equilibrium dialysis ligand binding method, that NPR1 itself is a SA receptor. However, Fu et al. (2012) found that the two NPR1-related proteins, NPR3 and NPR4, but not NPR1, bind to SA directly in conventional ligand binding assays and function as truly SA receptors. NPR3 and NPR4 are BTB-CUL3 ligases that direct the degradation of NPR1 via the 26S proteasome (Fu et al., 2012). Consistently, a *npr3npr4 *double mutant in *Arabidopsis *exhibits enhanced disease resistance, a phenotype that is opposite to that of the *npr1 *mutant (Fu et al., 2012). NPR1 is a master transcriptional positive co-activator of the TGA clade of bZIP (basic region/ leucine zipper motif) transcription factors controlling SA signaling and a large set of defense-related genes such as *PR* (*PATHOGENESIS-RELATED*) genes (Delaney et al., 1995; Dong, 2004). *PR* genes are a diverse group, but several encode proteins with antimicrobial activity (van Loon et al., 2006). NPR1 exists in at least two forms in the cell. When SA levels are low (e.g., in the absence of pathogen infection), NPR1 is sequestered in the cytoplasm as an oligomer through intermolecular disulphide bonds by *S*-nitrosylation of NPR1 via *S-*nitrosoglutathione activity (Tada et al., 2008). However, when the SA levels are high (e.g., after pathogen infection), redox changes in the cytosol trigger the monomerization of NPR1 by the activity of the thioredoxinsTRX-H3 and TRX-H5 (Tada et al., 2008). NPR1 monomers enter the nucleus via nuclear pore proteins, such as MODIFIER OF *snc1 *(MOS) 3, 6, and 7 (Cheng et al., 2009). In the nucleus, NPR1 bind TGA TFs initiating the SA-associated global transcriptional response (Dong, 2004). Strikingly, Fu et al. (2012) made interesting observations with crucial biological consequences for the establishment of plant immunity. NPR3 and NPR4 differ in their affinity for the SA hormone and in their roles in NPR1 degradation. NPR3 mediates NPR1 breakdown via 26S proteasome only in the presence of SA and NPR4 only in its absence (Fu et al., 2012). Thus, in healthy plants where SA is not present, NPR4, as part of the CUL3–NPR4 ubiquitin ligase, interacts with NPR1 to remove the NPR1 protein preventing the activation of energy-consuming defenses. In infected tissue, SA levels increase to high concentrations and promotes interaction of NPR3 with NPR1 to mediate degradation of NPR1, leading to strong defense-associated cell death at the site of attack. Upon infection, SA levels also increase at distal parts of plants. In these tissues, NPR1–NPR3 and NPR1–NPR4 interactions are both weakened, resulting in accumulation of NPR1, expression of defense genes without cell death and establishment of systemic acquired resistance. The recent identification of the SA receptors reveals how the hormone controls cell death and survival during plant immune responses in tissues close to and distant from the site of infection.

### ANTAGONISTIC CROSSTALK BETWEEN JA AND SA: SELECTING THE RIGHT PATHWAY

The activation of plant defenses implies allocation and ecological costs (Pieterse et al., 2012). For example, the allocation of resources to defense against one type of attacker can reduce the ability of the plant to respond to the challenge of a different invader. Thus, the antagonistic interplay between SA and JA seems to optimize the immune response against a specific single attacker. Plants infected by SA-inducing biotrophic pathogens often suppress JA-dependent defenses, apparently prioritizing the investment of resources in SA-dependent defense over JA-dependent responses (Spoel et al., 2007). Similarly, the elicitation of the JA pathway by pathogens can repress the SA response (Uppalapati et al., 2007). Recently, Van der Does et al. (2013) showed that SA suppresses JA signaling downstream of the COI1-JAZ receptor complex by targeting GCC-box motifs in JA-responsive promoters via a negative effect on the accumulation of the APETALA2/ETHYLENE RESPONSE FACTOR (AP2/ERF)-type transcriptional activator ORA59 (OCTADECANOID-RESPONSIVE ARABIDOPSIS59). Indeed, the GCC-box motif is overrepresented in JA-responsive promoters that are suppressed by SA and this promoter motif is sufficient for SA-mediated suppression of JA-induced gene expression (Van der Does et al., 2013). Interestingly, SA reduces the accumulation of the GCC-box binding TF ORA59, indicating that the antagonistic effect of SA on JA signaling is controlled at the level of transcriptional regulation, through the modulation of TF levels.

Several other proteins are known to play a role in regulating SA-mediated suppression of the JA pathway including mitogen-activated protein kinases, redox regulators, NPR1, and nuclear TGA and WRKY TFs among others (Pieterse et al., 2012). In *Arabidopsis*, mitogen activated protein kinase 4 (MPK4) acts as a negative regulator of SA signaling and positive regulator of JA signaling (Petersen et al., 2000; Brodersen et al., 2006). The *Arabidopsis* *mpk4* mutants show elevated SA levels, constitutive expression of SA responsive *PR* genes and increased resistance to *Pseudomonas syringae*. The expression of JA responsive genes and the resistance to *Alternaria brassicicola* is also impaired in *mpk4* mutants (Petersen et al., 2000; Brodersen et al., 2006).

Other important regulators affecting the antagonism between SA and JA-mediated signaling are glutaredoxins (GRXs), including GRX480 and several others of the ROXY class (Ndamukong et al., 2007; Zander et al., 2012). These proteins are central players in mediating redox regulation of protein activity because of their capacity to catalyze disulfide transitions (Meyer, 2008). These GRXs interact with TGA TFs involved in the regulation of SA responsive *PR* genes and antagonize JA-responsible genes such as *PLANT DEFENSIN1.2 *(*PDF1.2*) and *ORA59* (Ndamukong et al., 2007; Zander et al., 2012). For example, GRX480 interacts with TGA2 and TGA6 (Ndamukong et al., 2007) and has been implicated in SA-mediated suppression of the JA pathway. Indeed, *tga256 *triple and *tga2356 *quadruple mutants are impaired in SA-mediated suppression of the JA pathway (Ndamukong et al., 2007; Zander et al., 2009), indicating that TGAs effectively regulate SA–JA crosstalk.

Another important regulatory component is the SA master regulator NPR1 itself, which interacts with TGA TFs that are involved in the activation of SA-responsive PR genes (Dong, 2004). Nuclear localization of NPR1 is essential for SA-responsive defense gene expression, but not for SA-mediated suppression of the JA pathway (Spoel et al., 2003), indicating that SA–JA crosstalk is likely mediated by cytosolic NPR1. Despite this, NPR1 regulates several SA-dependent nuclear TFs or cofactors required for suppression of JA-gene expression such as TGA and WRKY TFs (Robert-Seilaniantz et al., 2011).

WRKY TFs are also an important node of convergence between SA and JA signaling (Pieterse et al., 2012). These include WRKY50, WRKY51, WRKY70, and WRKY62 among others (Pieterse et al., 2012). For example, overexpression of WRKY70 resulted in the constitutive expression of SA-responsive PR genes and enhanced resistance to the biotrophic pathogen *Erysiphe cichoracearum* but repressed the expression of JA-responsive marker gene *PDF1.2* and compromised resistance to the necrotrophic pathogen *Alternaria brassicicola* (Li et al., 2004; Li et al., 2006).

Finally, the JA nuclear TF MYC2 acts as a negative regulator of SA signaling in *Arabidopsis* as *myc2 *mutants show increased accumulation of SA, enhance expression of PR genes and increase resistance to *Pseudomonas syringae* compared to wild type plants (Laurie-Berry et al., 2006). Similarly, the triple mutant *myc2myc3myc4* in *Arabidopsis* is as resistant as *Arabidopsis* plants lacking the *COI1* gene to the bacterial pathogen *Pseudomonas syringae* (Fernandez-Calvo et al., 2011). Despite the increasing knowledge of proteins playing a role in SA–JA crosstalk, how crosstalk occurs at molecular level remains largely to be elucidated.

## NETWORKING OF HORMONES IN PLANT IMMUNITY

Plants use other hormones to fine-tune immune responses built on the SA and JA defense pathways (Pieterse et al., 2012). Incredible progress has been done in the last 2 years in the understanding of how SA and JA routes interact at the molecular level with other hormonal pathways. Here, we review the most outstanding examples regarding nuclear fine-tuning of hormonal balances in plant immunity.

### CROSSTALK JA-ET: FINE-TUNNING SYNERGY OF DEFENSES AGAINST PATHOGENS

Ethylene is another important plant hormone that works together with JA to regulate defense against necrotrophic pathogens (Wang et al., 2002). ET is perceived by a group of membrane-located receptor proteins including ETR1 (ETHYLENE RESPONSE 1), ERS1 (ETHYLENE RESPONSE SENSOR 1), ETR2 (ETHYLENE RESPONSE 2), ERS2 (ETHYLENE RESPONSE SENSOR 2), and EIN4 (ETHYLENE INSENSITIVE 4; Bleecker et al., 1988; Ecker, 1995; Hua et al., 1998). In normal conditions, where the level of ET is usually low, the receptors act to suppress ET response by activating the downstream negative regulator raf-like kinase CTR1 (CONSTITUTIVE TRIPLE RESPONSE 1) through direct physical interaction (Clark et al., 1998). Downstream of CTR1 is ETHYLENE INSENSITIVE 2 (EIN2), which is an essential positive regulator of ET signaling (Alonso et al., 2003). CTR1 suppression is relieved upon ET binding to the trans-membrane domain of the receptors, facilitating subsequent activation of a diverse set of ET-responsive TFs downstream of EIN2 including EIN3 (ETHYLENE INSENSITIVE 3) and its nearest homolog EIL1 (EIN3-LIKE 1; Chao et al., 1997; Wang et al., 2006). EIN3 levels are regulated through the action of at least two related F-box proteins, EIN3-Binding F-box 1 (EBF1) and EBF2 which are thought to repress EIN3 levels when ET is low (Guo and Ecker, 2003; Potuschak et al., 2003; Binder et al., 2007). EIN3 and EIL1 belong to a multigene family of TFs including six putative members of this family in *Arabidopsis* (Wang et al., 2002). However, EIN3 and EIL1 TFs are largely responsible for primary gene induction downstream of ET sensing including defense against pathogens (Guo and Ecker, 2004).

The crosstalk between JA and ET can be rather complex and context-dependent. However, JA and ET signaling act synergistically during plant defense against necrothrophic pathogens (Broekaert et al., 2006). In *Arabidopsis*, two major branches of the JA signaling pathway are recognized, the MYC branch and the ERF branch (Lorenzo et al., 2004; Dombrecht et al., 2007). In general, the MYC branch is associated with the wound response and defense against insect herbivores (Lorenzo et al., 2004), whereas the ERF branch is associated with enhanced resistance to necrotrophic pathogens (Berrocal-Lobo et al., 2002; Lorenzo et al., 2003). The MYC branch is controlled by MYC-type TFs leading to expression of JA-responsive marker genes such as *VEGETATIVE STORAGE PROTEIN2* (*VSP2*). The ERF branch is regulated by members of the AP2/ERF family of TFs, such as ERF1 and ORA59 (Lorenzo et al., 2003; McGrath et al., 2005; Dombrecht et al., 2007), that regulate the expression of JA-responsive marker genes such as *PDF1.2*. Overexpression of *ERF1 *enhances resistance against* B. cinerea *and other necrothrophic pathogens, and increases susceptibility to the hemibiotroph *Pseudomonas syringe* (Berrocal-Lobo et al., 2002; Berrocal-Lobo and Molina, 2004). The ERF branch is synergistically regulated by the ET- and the JA-pathways, whereas they antagonize in the regulation of the MYC branch (Lorenzo and Solano, 2005). Zhou and colleagues recently showed that JAZ proteins interact directly with the ET TFs EIN3 and EIL1, inhibiting the transcriptional activity of these TFs by recruitment of the transcriptional co-repressor HISTONE DEACETYLASE6 (HDA6; Zhu et al., 2011). EIN3 and EIL1 transcriptional regulators are stabilized in the presence of ET, allowing the expression of ET-responsive genes. However, their binding to JAZ proteins partially represses the function of EIN3/EIL1, possibly by suppressing their DNA binding capacity. This provides a second level of transcriptional regulation throught JA (Zhu et al., 2011). In the presence of JA, EIN3/EIL1 TFs are released from the JAZ repression allowing full activity of these TFs. This results in the synergistic activation of *ERF1 *and its downstream target genes such as *PDF1.2* (Zhu et al., 2011). In addition, EIN3 and EIL1 are known repressors of *SID2* (SALICYLIC ACID INDUCTION DEFICIENT 2), a gene encoding an isochorismate synthase required for SA biosynthesis (Chen et al., 2009). Thus, it is possible that JAZ repressors also mediate JA–SA antagonistic crosstalk thought the suppression exerted on EIN3 and EIL1.

### CROSSTALK JA-GIBBERELLINS: BALANCING DEFENSE AND GROWTH

Gibberellins are plant hormones involved in the regulation of plant growth in response to endogenous and environmental signals (Sun, 2011). DELLA proteins are key components of GA signaling and nuclear localized negative regulators of plant growth-promoting TFs, such as PIFs (PHYTOCHROME INTERACTING FACTORS; de Lucas et al., 2008; Feng et al., 2008). In *Arabidopsis*, there are five DELLAs: GAI (GIBBERELLIC ACID INSENSITIVE), RGA (REPRESSOR OF GA1-3), RGL1 (RGA-like1), RGL2, and RGL3 (Peng et al., 1997; Silverstone et al., 2001; Lee et al., 2002; Tyler et al., 2004). Binding of GA to its receptor GID1 (GA INSENSITIVE DWARF1) promotes the GID1–DELLA interaction, which in turn stimulates the interaction between DELLAs and the specific E3 ubiquitin ligase SLY1/GID2 complex, leading to subsequent degradation of DELLAs by the 26S proteasome and activation of PIFs (Silverstone et al., 2001; Tyler et al., 2004; Harberd et al., 2009). Importantly, DELLAs modulate plant immune response by modulating the balance of JA/SA. For example, DELLA activity promotes plant resistance to necrotrophs by potentiating JA signaling and increases plant susceptibility to virulent biotrophs by attenuating the SA pathway (Navarro et al., 2008). Accordingly, JA mediated pathogen defense is attenuated in DELLA loss-of-function mutants while defense genes are hyperactivated by JA in constitutively active DELLA mutants (Navarro et al., 2008). Moreover, a GA-deficient mutant *ga1* shows upregulated expression of JA-responsive defense genes (Hou et al., 2010), indicating that DELLA proteins interact with JA signaling in a positive manner. Interestingly, it was recently reported that DELLA interact with JAZ proteins and modulate JA signaling via competitive binding with MYC TFs for engaging to JAZ repressors (Chen et al., 2004; Hou et al., 2010; Wild et al., 2012; Yang et al., 2012). Consistent with this model, MYC2-dependent JA-responsive genes are more induced in response to JA treatment in mutant backgrounds accumulating DELLAs such as the GA biosynthetic mutant *ga1-3* compared to mock treatment controls (Hou et al., 2010). Indeed, overexpression of RGL3 activates MYC2-dependent JA-induced gene expression, whereas *rgl3* mutation reduces it (Wild et al., 2012). Consistently, RGL3 positively regulates JA-mediated resistance to the necrotroph *B. cinerea* and susceptibility to the hemibiotroph *Pseudomonas syringae *(Wild et al., 2012). On the other hand, Yang et al. (2012) recently reported that JA prioritizes defense over growth by interfering with GAs signaling cascade through the COI1–JAZ–DELLA–PIF signaling module. This provides an explanation about why activation of defense in plants is often accompanied by a significant inhibition of growth. Thus, DELLAs and JAZs seem to integrate environmental signals that enable plants to adapt their growth and development according to their surrounding environment.

### CROSSTALK SA/CK: GROWTH HORMONES REGULATING PLANT IMMUNITY

Cytokinin are growth control hormones, which promote cell division, nutrient mobilization, and leaf longevity (Choi et al., 2011). CK have recently emerged as modulators of plant immunity (Choi et al., 2011). In *Arabidopsis*, hybrid histidine protein kinases (AHKs) serve as CK receptors (Inoue et al., 2001; Suzuki et al., 2001; Ueguchi et al., 2001; Yamada et al., 2001). Histidine phosphotransfer proteins (AHPs) transmit the signal from AHKs to nuclear response regulators (ARRs), which can activate or repress transcription (Hwang et al., 2012). CKs promote resistance of *Arabidopsis* to *Pseudomonas syringae*, which correlates with increased SA biosynthesis and *PR1* expression (Choi et al., 2010). As shown by Choi et al. (2010), this is probably mediated by a direct interaction between ARR2, a TF involved in CK signaling, and the SA response TF TGA3. ARR2 specifically interacts with TGA3 and is recruited to the PR1 promoter, inducing resistance to *Pseudomonas syringae*. In contrast, the alternative ARR1 related factor that cannot interact with TGA3, fails to induce resistance to *Pseudomonas syringae *(Choi et al., 2010, 2011). Moreover, the SA biosynthetic genes, *SID1* and *SID2*, and the SA-responsive genes, PR1 and PR5, are over-induced in 35S:ARR2 plants compared to Col-0 controls inoculated with *Pseudomonas syringae *(Choi et al., 2010). Therefore, crosstalk SA/CK is based on the regulation of a module in the SA-mediated defense response network by the ARR2 transcriptional factor.

## HIJACKING NUCLEAR HORMONAL NETWORKS BY MICROBES

Microbial pathogens have also developed the ability to manipulate the defense-related regulatory network of plant hormones to cause hormonal imbalances and inappropriate activation of defense responses for their own benefit (Robert-Seilaniantz et al., 2011). In recent years, there have been a number of examples of plant pathogens that hijack specific hormone-regulated signaling pathways to redirect the immune response in the nucleus. Microbes do this by producing plant hormones, phytohormone mimics, or virulence effectors that target hormone signaling components. Here, we describe the best characterized examples at the molecular level of specific nuclear hormonal manipulation by microbes, which exemplify the ingenious ways by which pathogens can take control over the plant’s hormone signaling network to suppress host immunity.

### PRODUCTION OF PLANT HORMONES AND HORMONE MIMICS BY PATHOGENS

Interestingly, many pathogens are capable of synthesizing phytohormones. Different bacterial or fungal species are known to produce JA (Mittal and Davis, 1995), ET (Weingart and Volksch, 1997; Weingart et al., 2001), CK (Kakimoto, 2003), ABA (Kitagawa et al., 1995; Siewers et al., 2006), and auxins (Spaepen et al., 2007). For example, the necrotrophic fungi *B. cinerea *produces ABA, CK and secretes an exopolysaccharide that acts as an elicitor of the SA pathway (El Oirdi et al., 2011; Robert-Seilaniantz et al., 2011). In contrast, *Fusarium oxysporum *produces only ABA (Robert-Seilaniantz et al., 2011). Biotrophic fungi such as *Cladosporium fulvum*, *Ustilago maydis*, *Pyrenopeziza brassicae*, and *Venturia inaequalis *are well known to produce CKs (Robert-Seilaniantz et al., 2011). Moreover, the biotrophic bacterial pathogen *Ralstonia solanacearum *produce ET and indolic compounds related to auxin (Valls et al., 2006), and *Pseudomonas syringae *pv. *syringae* B728a also produces auxin by converting host indole acetonitrile into IAA using a nitrilase (Howden et al., 2009). Therefore, it is not surprising that some pathogens trigger symptoms indicative of hormonal imbalances. This is the case of *Agrobacterium tumefaciens* that induces gall formation following T-DNA transfer and *in planta* production of auxin and CK (Akiyoshi et al., 1983). The increased height of rice seedling infected with* Gibberella fujikuroi *is a consequence of production of GAs by the pathogen (Robert-Seilaniantz et al., 2011). Despite all these examples, the roles of hormones during plant–pathogen interactions are still not fully understood in most cases.

Probably, the best understood example corresponds to the production of coronatine (COR), a mimic of the bioactive jasmonate hormone JA-Ile, by some strains of *Pseudomonas syringae* (Brooks et al., 2004; Fonseca et al., 2009b). COR, as the JA-Ile hormone, is perceived in the nucleus through the COI1/JAZ receptor complex that upon COR binding triggers the degradation of JAZ transcriptional repressors via the 26S proteasome (Chini et al., 2007; Thines et al., 2007; Sheard et al., 2010). This leads to de-repression of JAZ-interacting TFs that initiate the transcription of JA-dependent genes, further inhibiting SA-dependent defenses against the bacteria. Indeed, COR is more active than the own JA-Ile plant hormone itself in triggering the COI1–JAZ complexes formation and subsequent JAZ degradation (Katsir et al., 2008; Fonseca et al., 2009b), indicating than COR acts as a potent virulence factor in plants. COR contributes to disease symptomatology by inducing chlorotic lesions (Kloek et al., 2001; Brooks et al., 2004; Uppalapati et al., 2007), facilitates entry of the bacteria into the plant host by stimulating the opening of stomata (Melotto et al., 2006, 2008) and promotes bacterial growth by inhibiting SA-dependent defenses required for *Pseudomonas syringae* resistance through the activation of its antagonistic JA pathway (Cui et al., 2005; Laurie-Berry et al., 2006). Interestingly, it was recently reported that COR suppresses a SA-independent pathway contributing to callose deposition, a hallmark of plant resistance, by reducing accumulation of an indole glucosinolate in a COI1-independent manner (Geng et al., 2012). This indicates that COR may have additional targets to the COI1/JAZ receptor complex inside plant cells opening novel and interesting areas of research. Thus, acquisition of COR by these *Pseudomonas* pathogens has been of tremendous adaptative importance during host-pathogen evolution because it has allowed bacteria to manipulate the host hormonal network to promote susceptibility.

### PATHOGEN EFFECTORS TARGETING NUCLEAR HORMONE SIGNALING COMPONENTS

In addition to producing hormones themselves, many pathogens also introduce into the plant cell an arsenal of virulence effector proteins (Jones and Dangl, 2006). Within the cell, these effectors interact with host proteins to promote pathogenesis (Jones and Dangl, 2006). Several bacterial effectors are known to affect hormonal equilibrium into the plant cell. For instance, the *Pseudomonas* effector AvrPtoB stimulates ABA biosynthesis and ABA responses, which in turn antagonize SA biosynthesis and SA-mediated defenses (de Torres Zabala et al., 2007, 2009). In another example, the *Pseudomonas* effector AvrRpt2 alters the auxin physiology to promote disease (Chen et al., 2007). However, how these effectors are able to impact in the hormonal homeostasis remains a mystery. In contrast to the above examples, the transcription activators-like (TAL) effectors of *Xanthomonas* spp. are a reference in their mode of action showing extreme target specificity (Boch and Bonas, 2010). This class of effectors is exemplified by AvrBs3 which is imported into the plant cell nucleus, and targeted to effector-specific gene promoters by mimicking eukaryotic TFs (Boch and Bonas, 2010). The specificity of these TFs arises from interactions between the DNA binding domain of each effector and a sequence in the target gene promoter called the *UPA* box. In a stunning series of papers, the molecular basis of promoter recognition by TAL effectors was decoded (Kay et al., 2007; Boch et al., 2009). The DNA binding domain comprises a central tandem repeat region (Kay et al., 2007; Boch et al., 2009; Moscou and Bogdanove, 2009). Strikingly, two hypervariable amino acid residues in each repeat specify interaction with a characteristic nucleotide within the effector recognition site (Boch and Bonas, 2010). Thus, the nucleotide sequence of the target DNA can be predicted with complete accuracy based on the amino acid sequence of the tandem repeat domain. Interestingly, auxin-induced genes and α-expansins are among the *UPA *genes regulated by AvrBs3 (Kay et al., 2007). Indeed, AvrBs3 directly targets *UPA20*, a bHLH TF that controls cell enlargement and plant cell hypertrophy phenotype through the activation of putative α-expansin UPA7 which is involved in cell wall softening (Kay et al., 2007). Consistently, AvrBs3 causes tissue hypertrophy, which is due to an enlargement of the mesophyll cells in infected tissue and resemble symptoms indicative of hormonal imbalances. This might help the bacteria to escape from infection sites to facilitate bacterial spreading.

Recently, it has been reported that the *Xanthomonas campestris* effector XopD is able to target MYB30, a TF that positively regulates *Arabidopsis* defense and associated cell death responses to bacteria through transcriptional activation of genes related to very-long-chain fatty acid (VLCFA) metabolism (Canonne et al., 2011). XopD specifically interacts with MYB30, resulting in inhibition of the MYB30-dependent transcriptional activation of VLCFA-related genes and suppression of *Arabidopsis* defense (Canonne et al., 2011; Canonne and Rivas, 2012). Interestingly, it was previously reported that MYB30 is a direct target of BES1, a key regulator of BR signaling, and cooperates with BES1 to regulate BR-induced gene expression (Li et al., 2009). However, whether XopD suppresses immunity by also affecting BR homeostasis is currently unknown.

### NUCLEAR MODULATION OF HORMONAL PATHWAYS BY BENEFICIAL MICROBES

Beneficial microbes interacting with plants establish long-term relationships with their hosts to fulfill their life cycles (Zamioudis and Pieterse, 2012). In order to do this, they need to contend with the defense mechanisms of the plant to develop within the host and feed on living cells. Recently, the signals from two beneficial microbes that mediate symbiosis with their host plants have been characterized and results nicely show that in both cases these beneficial effectors hijack hormone signaling at the nucleus (Kloppholz et al., 2011; Plett et al., 2011). The MYCORRHIZAL INDUCED SMALL SECRETED PROTEIN 7 (MiSSP7), the most highly symbiosis-upregulated gene from the ectomycorrhizal fungus *Laccaria bicolor*, encodes an effector protein indispensable for the establishment of mutualism with their host plants. MiSSP7 is secreted by the fungus upon receipt of diffusible signals from plant roots, imported into the plant cell via phosphatidylinositol-3-phosphate-mediated endocytosis, and targeted to the plant nucleus where it alters the transcriptome of the plant cell promoting the expression of auxin-responsible genes (Plett et al., 2011).

The SP7 effector of the fungus *Glomus intraradices *is another example of symbiotic effectors that promote a biotrophic interaction by affecting hormonal signaling pathways (Kloppholz et al., 2011). SP7 possess immune-suppressive function by targeting the ET signaling pathway, which is an important component of plant immune responses in the roots (Boutrot et al., 2010). The role of SP7 in hijacking ET signaling is interesting as recent work indicates that the ET pathway is a key determinant in the colonization of plant tissues by fungus (Splivallo et al., 2009; Camehl et al., 2010). SP7 localizes to the plant nucleus where it interacts with ET response factor 19 (ERF19) to repress plant defense signaling. *ERF19* is highly induced in roots by the fungal pathogen *Colletotrichum trifolii* as well as by several fungal extracts, but only transiently during mycorrhiza colonization. When constitutively expressed in roots, SP7 leads to higher mycorrhization while reducing the levels of *Colletotrichum trifolii*-mediated defense responses (Kloppholz et al., 2011). Thus, beneficial microbes also contain effectors that resemble those of pathogenic fungi, nematodes, and bacteria. These effectors are similarly targeted to the plant nucleus to manipulate hormonal signaling and colonization of the plant tissues, and thus can be considered a mutualism effector.

## CONCLUDING REMARKS

These are exciting times for hormonal signaling research as recent major discoveries have been made in the last few years. Recent knowledge regarding the perception of plant hormones and the involvement of specific hormone-related proteins in direct cross-talk between various hormonal and environmental signals has advanced our understanding of the molecular basis of how several hormones control plant immunity to a broad range of different pathogens. The recent findings suggest that several important mediators of hormone cross-talk are transcriptional factors or repressors, indicating that cross-talk predominantly takes place in the nucleus downstream of signal transduction, at the level of gene transcription. JAZs and DELLAs are all repressors of positive transcriptional regulators of hormone signaling. The rapid removal of hormonal transcriptional repressors through 26S proteasome and the ability to interact with each other (and additional TFs associated to other hormonal pathways such as EIN3/EIL1) provides a paradigm to explain the rapid and appropriate level of defense responses to specific signals. Moreover, key hormones regulating growth, such as GA and auxins, are involved in the orchestration of the plant immune response suggesting that developmental and defense signaling networks are closely interconnected. This would explain why activation of costly defenses is often accompanied by significant growth inhibition. Thus, hormone homeostasis and cross-talk seem to be a dominant feature to maximize defenses and to fine-tune growth and protection. Not surprisingly, pathogens and beneficial microbes have learned the necessity of manipulation of plant hormonal pathways to rewire the immune signaling circuitry for their own benefit. Indeed, the importance of hormones in plant immunity is highlighted by the increasing number of pathogens that are predicted to produce phytohormones or phytohormone mimics, and the recent findings indicating that microbial effectors also targets hormonal pathways to promote disease or establish beneficial interactions (Robert-Seilaniantz et al., 2011). Due to the increasing number of effectors known to be targeted to the plant cell nucleus (Schornack et al., 2010; Canonne and Rivas, 2012), it is plausible to expect direct virulence activities on hormonal components in order to subvert host transcription. An improved understanding of the mechanisms by which pathogens use their toxins and effectors to manipulate and target hormonal components controlling immunity in plants will prove invaluable for identifying defensive hubs in plants controlling immunity and developing plant lines with improved resistance. It truly is an exciting time to start understanding how complex hormonal signaling and interactions are translated into a definite coordinated defense response that is effective against the type of pathogen that the plant is encountering.

## Conflict of Interest Statement

The authors declare that the research was conducted in the absence of any commercial or financial relationships that could be construed as a potential conflict of interest.

## References

[B1] Akiyoshi D. E. Morris R. O. Hinz R. Mischke B. S. Kosuge T. Garfinkel D. J. (1983). Cytokinin/auxin balance in crown gall tumors is regulated by specific loci in the T-DNA. *Proc. Natl. Acad. Sci. U.S.A.* 80 407–411 16593270 10.1073/pnas.80.2.407 PMC393386

[B2] Alonso J. M. Stepanova A. N. Solano R. Wisman E. Ferrari S. Ausubel F. M. (2003). Five components of the ethylene-response pathway identified in a screen for weak ethylene-insensitive mutants in *Arabidopsis*. *Proc. Natl. Acad. Sci. U.S.A.* 100 2992–2997 12606727 10.1073/pnas.0438070100 PMC151454

[B3] An C. Mou Z. (2011). Salicylic acid and its function in plant immunity. *J. Integr. Plant Biol.* 53 412–428 21535470 10.1111/j.1744-7909.2011.01043.x

[B4] Berrocal-Lobo M. Molina A. (2004). Ethylene response factor 1 mediates *Arabidopsis* resistance to the soilborne fungus *Fusarium oxysporum*. *Mol. Plant Microbe Interact.* 17 763–770 15242170 10.1094/MPMI.2004.17.7.763

[B5] Berrocal-Lobo M. Molina A. Solano R. (2002). Constitutive expression of ETHYLENE-RESPONSE-FACTOR1 in *Arabidopsis* confers resistance to several necrotrophic fungi. *Plant J.* 29 23–32 12060224 10.1046/j.1365-313x.2002.01191.x

[B6] Binder B. M. Walker J. M. Gagne J. M. Emborg T. J. Hemmann G. Bleecker A. B. (2007). The *Arabidopsis* EIN3 binding F-Box proteins EBF1 and EBF2 have distinct but overlapping roles in ethylene signaling. *Plant Cell* 19 509–523 17307926 10.1105/tpc.106.048140 PMC1867343

[B7] Bleecker A. B. Estelle M. A. Somerville C. Kende H. (1988). Insensitivity to ethylene conferred by a dominant mutation in *Arabidopsis thaliana*. *Science* 241 1086–1089 17747490 10.1126/science.241.4869.1086

[B8] Boch J. Bonas U. (2010). *Xanthomonas* AvrBs3 family-type III effectors: discovery and function. *Annu. Rev. Phytopathol.* 48 419–436 19400638 10.1146/annurev-phyto-080508-081936

[B9] Boch J. Scholze H. Schornack S. Landgraf A. Hahn S. Kay S. (2009). Breaking the code of DNA binding specificity of TAL-type III effectors. *Science* 326 1509–1512 19933107 10.1126/science.1178811

[B10] Boutrot F. Segonzac C. Chang K. N. Qiao H. Ecker J. R. Zipfel C. (2010). Direct transcriptional control of the *Arabidopsis* immune receptor FLS2 by the ethylene-dependent transcription factors EIN3 and EIL1. *Proc. Natl. Acad. Sci. U.S.A.* 107 14502–14507 20663954 10.1073/pnas.1003347107 PMC2922558

[B11] Broekaert W. F. Delaure S. L. De Bolle M. F. Cammue B. P. (2006). The role of ethylene in host-pathogen interactions. *Annu. Rev. Phytopathol.* 44 393–416 16602950 10.1146/annurev.phyto.44.070505.143440

[B12] Brodersen P. Petersen M. Bjorn Nielsen H. Zhu S. Newman M. A. Shokat K. M. (2006). *Arabidopsis* MAP kinase 4 regulates salicylic acid- and jasmonic acid/ethylene-dependent responses via EDS1 and PAD4. *Plant J.* 47 532–546 16813576 10.1111/j.1365-313X.2006.02806.x

[B13] Brooks D. M. Hernandez-Guzman G. Kloek A. P. Alarcon-Chaidez F. Sreedharan A. Rangaswamy V. (2004). Identification and characterization of a well-defined series of coronatine biosynthetic mutants of *Pseudomonas syringae* pv. tomato DC3000. *Mol. Plant Microbe Interact.* 17 162–174 14964530 10.1094/MPMI.2004.17.2.162

[B14] Camehl I. Sherameti I. Venus Y. Bethke G. Varma A. Lee J. (2010). Ethylene signalling and ethylene-targeted transcription factors are required to balance beneficial and nonbeneficial traits in the symbiosis between the endophytic fungus *Piriformospora indica* and *Arabidopsis thaliana*. *New Phytol.* 185 1062–1073 20085621 10.1111/j.1469-8137.2009.03149.x

[B15] Canonne J. Marino D. Jauneau A. Pouzet C. Briere C. Roby D. (2011). The *Xanthomonas* type III effector XopD targets the *Arabidopsis* transcription factor MYB30 to suppress plant defense. *Plant Cell* 23 3498–3511 21917550 10.1105/tpc.111.088815 PMC3203416

[B16] Canonne J. Rivas S. (2012). Bacterial effectors target the plant cell nucleus to subvert host transcription. *Plant Signal. Behav.* 7 217–221 22353865 10.4161/psb.18885 PMC3405691

[B17] Chao Q. Rothenberg M. Solano R. Roman G. Terzaghi W. Ecker J. R. (1997). Activation of the ethylene gas response pathway in *Arabidopsis* by the nuclear protein ETHYLENE-INSENSITIVE3 and related proteins. *Cell* 89 1133–1144 9215635 10.1016/s0092-8674(00)80300-1

[B18] Chen H. Mccaig B. C. Melotto M. He S. Y. Howe G. A. (2004). Regulation of plant arginase by wounding, jasmonate, and the phytotoxin coronatine. *J. Biol. Chem.* 279 45998–46007 15322128 10.1074/jbc.M407151200

[B19] Chen H. Xue L. Chintamanani S. Germain H. Lin H. Cui H. (2009). ETHYLENE INSENSITIVE3 and ETHYLENE INSENSITIVE3-LIKE1 repress SALICYLIC ACID INDUCTION DEFICIENT2 expression to negatively regulate plant innate immunity in *Arabidopsis*. *Plant Cell* 21 2527–2540 19717619 10.1105/tpc.108.065193 PMC2751940

[B20] Cheng Y. T. Germain H. Wiermer M. Bi D. Xu F. Garcia A. V. (2009). Nuclear pore complex component MOS7/Nup88 is required for innate immunity and nuclear accumulation of defense regulators in *Arabidopsis*. *Plant Cell* 21 2503–2516 19700630 10.1105/tpc.108.064519 PMC2751965

[B21] Cheng Z. Sun L. Qi T. Zhang B. Peng W. Liu Y. (2011). The bHLH transcription factor MYC3 interacts with the Jasmonate ZIM-domain proteins to mediate jasmonate response in *Arabidopsis*. *Mol. Plant* 4 279–288 21242320 10.1093/mp/ssq073

[B22] Chen Z. Agnew J. L. Cohen J. D. He P. Shan L. Sheen J. (2007). *Pseudomonas syringae* type III effector AvrRpt2 alters *Arabidopsis thaliana* auxin physiology. *Proc. Natl. Acad. Sci. U.S.A.* 104 20131–20136 18056646 10.1073/pnas.0704901104 PMC2148434

[B23] Chini A. Boter M. Solano R. (2009a). Plant oxylipins: COI1/JAZs/MYC2 as the core jasmonic acid-signalling module. *FEBS J.* 276 4682–4692 19663905 10.1111/j.1742-4658.2009.07194.x

[B24] Chini A. Fonseca S. Chico J. M. Fernandez-Calvo P. Solano R. (2009b). The ZIM domain mediates homo- and heteromeric interactions between *Arabidopsis* JAZ proteins. *Plant J.* 59 77–87 19309455 10.1111/j.1365-313X.2009.03852.x

[B25] Chini A. Fonseca S. Fernandez G. Adie B. Chico J. M. Lorenzo O. (2007). The JAZ family of repressors is the missing link in jasmonate signalling. *Nature* 448 666–671 17637675 10.1038/nature06006

[B26] Choi J. Choi D. Lee S. Ryu C. M. Hwang I. (2011). Cytokinins and plant immunity: old foes or new friends? *Trends Plant Sci*. 16 388–394 21470894 10.1016/j.tplants.2011.03.003

[B27] Choi J. Huh S. U. Kojima M. Sakakibara H. Paek K. H. Hwang I. (2010). The cytokinin-activated transcription factor ARR2 promotes plant immunity via TGA3/NPR1-dependent salicylic acid signaling in *Arabidopsis*. *Dev. Cell* 19 284–295 20708590 10.1016/j.devcel.2010.07.011

[B28] Clark K. L. Larsen P. B. Wang X. Chang C. (1998). Association of the *Arabidopsis* CTR1 Raf-like kinase with the ETR1 and ERS ethylene receptors. *Proc. Natl. Acad. Sci. U.S.A.* 95 5401–5406 9560288 10.1073/pnas.95.9.5401 PMC20273

[B29] Cui J. Bahrami A. K. Pringle E. G. Hernandez-Guzman G. Bender C. L. Pierce N. E. (2005). *Pseudomonas syringae* manipulates systemic plant defenses against pathogens and herbivores. *Proc. Natl. Acad. Sci. U.S.A.* 102 1791–1796 15657122 10.1073/pnas.0409450102 PMC547856

[B30] Delaney T. P. Friedrich L. Ryals J. A. (1995). *Arabidopsis* signal transduction mutant defective in chemically and biologically induced disease resistance. *Proc. Natl. Acad. Sci. U.S.A.* 92 6602–6606 11607555 10.1073/pnas.92.14.6602 PMC41566

[B31] de Lucas M. Daviere J. M. Rodriguez-Falcon M. Pontin M. Iglesias-Pedraz J. M. Lorrain S. (2008). A molecular framework for light and gibberellin control of cell elongation. *Nature* 451 480–484 18216857 10.1038/nature06520

[B32] de Torres Zabala M. Bennett M. H. Truman W. H. Grant M. R. (2009). Antagonism between salicylic and abscisic acid reflects early host-pathogen conflict and moulds plant defence responses. *Plant J.* 59 375–386 19392690 10.1111/j.1365-313X.2009.03875.x

[B33] de Torres Zabala M. Truman W. Bennett M. H. Lafforgue G. Mansfield J. W. Rodriguez Egea P. (2007). *Pseudomonas syringae* pv. tomato hijacks the* Arabidopsis* abscisic acid signalling pathway to cause disease. *EMBO J.* 26 1434–1443 17304219 10.1038/sj.emboj.7601575 PMC1817624

[B34] De Vos M. Van Oosten V. R. Van Poecke R. M. Van Pelt J. A. Pozo M. J. Mueller M. J. (2005). Signal signature and transcriptome changes of *Arabidopsis* during pathogen and insect attack. *Mol. Plant Microbe Interact.* 18 923–937 16167763 10.1094/MPMI-18-0923

[B35] Dombrecht B. Xue G. P. Sprague S. J. Kirkegaard J. A. Ross J. J. Reid J. B. (2007). MYC2 differentially modulates diverse jasmonate-dependent functions in *Arabidopsis*. *Plant Cell* 19 2225–2245 17616737 10.1105/tpc.106.048017 PMC1955694

[B36] Dong X. (2004). NPR1, all things considered. *Curr. Opin. Plant Biol.* 7 547–552 15337097 10.1016/j.pbi.2004.07.005

[B37] Ecker J. R. (1995). The ethylene signal transduction pathway in plants. *Science* 268 667–675 7732375 10.1126/science.7732375

[B38] El Oirdi M. El Rahman T. A. Rigano L. El Hadrami A. Rodriguez M. C. Daayf F. (2011). *Botrytis cinerea* manipulates the antagonistic effects between immune pathways to promote disease development in tomato. *Plant Cell* 23 2405–2421 21665999 10.1105/tpc.111.083394 PMC3160041

[B39] Feng S. Martinez C. Gusmaroli G. Wang Y. Zhou J. Wang F. (2008). Coordinated regulation of *Arabidopsis thaliana* development by light and gibberellins. *Nature* 451 475–479 18216856 10.1038/nature06448 PMC2562044

[B40] Fernandez-Calvo P. Chini A. Fernandez-Barbero G. Chico J. M. Gimenez-Ibanez S. Geerinck J. (2011). The *Arabidopsis* bHLH transcription factors MYC3 and MYC4 are targets of JAZ repressors and act additively with MYC2 in the activation of jasmonate responses. *Plant Cell* 23 701–715 21335373 10.1105/tpc.110.080788 PMC3077776

[B41] Feys B. Benedetti C. E. Penfold C. N. Turner J. G. (1994). *Arabidopsis* mutants selected for resistance to the phytotoxin coronatine are male sterile, insensitive to methyl jasmonate, and resistant to a bacterial pathogen. *Plant Cell* 6 751–759 12244256 10.1105/tpc.6.5.751 PMC160473

[B42] Fonseca S. Chico J. M. Solano R. (2009a). The jasmonate pathway: the ligand, the receptor and the core signalling module. *Curr. Opin. Plant Biol.* 12 539–547 19716757 10.1016/j.pbi.2009.07.013

[B43] Fonseca S. Chini A. Hamberg M. Adie B. Porzel A. Kramell R. (2009b). (+)-7-iso-Jasmonoyl-L-isoleucine is the endogenous bioactive jasmonate. *Nat. Chem. Biol.* 5 344–350 19349968 10.1038/nchembio.161

[B44] Fu Z. Q. Yan S. Saleh A. Wang W. Ruble J. Oka N. (2012). NPR3 and NPR4 are receptors for the immune signal salicylic acid in plants. *Nature* 486 228–232 22699612 10.1038/nature11162 PMC3376392

[B45] Geng X. Cheng J. Gangadharan A. Mackey D. (2012). The coronatine toxin of *Pseudomonas syringae* is a multifunctional suppressor of *Arabidopsis* defense. *Plant Cell* 24 4763–4774 23204405 10.1105/tpc.112.105312 PMC3531865

[B46] Glazebrook J. (2005). Contrasting mechanisms of defense against biotrophic and necrotrophic pathogens. *Annu. Rev. Phytopathol.* 43 205–227 16078883 10.1146/annurev.phyto.43.040204.135923

[B47] Grant M. Lamb C. (2006). Systemic immunity. *Curr. Opin. Plant Biol.* 9 414–420 16753329 10.1016/j.pbi.2006.05.013

[B48] Guo H. Ecker J. R. (2003). Plant responses to ethylene gas are mediated by SCF(EBF1/EBF2)-dependent proteolysis of EIN3 transcription factor. *Cell* 115 667–677 14675532 10.1016/s0092-8674(03)00969-3

[B49] Guo H. Ecker J. R. (2004). The ethylene signaling pathway: new insights. *Curr. Opin. Plant Biol.* 7 40–49 14732440 10.1016/j.pbi.2003.11.011

[B50] Harberd N. P. Belfield E. Yasumura Y. (2009). The angiosperm gibberellin-GID1-DELLA growth regulatory mechanism: how an “inhibitor of an inhibitor” enables flexible response to fluctuating environments. *Plant Cell* 21 1328–1339 19470587 10.1105/tpc.109.066969 PMC2700538

[B51] Hou X. Lee L. Y. Xia K. Yan Y. Yu H. (2010). DELLAs modulate jasmonate signaling via competitive binding to JAZs. *Dev. Cell* 19 884–894 21145503 10.1016/j.devcel.2010.10.024

[B52] Howden A. J. Rico A. Mentlak T. Miguet L. Preston G. M. (2009). *Pseudomonas syringae* pv. *syringae* B728a hydrolyses indole-3-acetonitrile to the plant hormone indole-3-acetic acid. *Mol. Plant Pathol*. 10 857–865 19849791 10.1111/j.1364-3703.2009.00595.x PMC6640395

[B53] Hua J. Sakai H. Nourizadeh S. Chen Q. G. Bleecker A. B. Ecker J. R. (1998). EIN4 and ERS2 are members of the putative ethylene receptor gene family in *Arabidopsis*. *Plant Cell* 10 1321–1332 9707532 10.1105/tpc.10.8.1321 PMC144061

[B54] Hwang I. Sheen J. Muller B. (2012). Cytokinin signaling networks. *Annu. Rev. Plant Biol.* 63 353–380 22554243 10.1146/annurev-arplant-042811-105503

[B55] Inoue T. Higuchi M. Hashimoto Y. Seki M. Kobayashi M. Kato T. (2001). Identification of CRE1 as a cytokinin receptor from *Arabidopsis*. *Nature* 409 1060–1063 11234017 10.1038/35059117

[B56] Jones J. D. Dangl J. L. (2006). The plant immune system. *Nature* 444 323–329 17108957 10.1038/nature05286

[B57] Kagale S. Links M. G. Rozwadowski K. (2010). Genome-wide analysis of ethylene-responsive element binding factor-associated amphiphilic repression motif-containing transcriptional regulators in *Arabidopsis*. *Plant Physiol.* 152 1109–1134 20097792 10.1104/pp.109.151704 PMC2832246

[B58] Kakimoto T. (2003). Biosynthesis of cytokinins. *J. Plant Res.* 116 233–239 12721785 10.1007/s10265-003-0095-5

[B59] Katsir L. Schilmiller A. L. Staswick P. E. He S. Y. Howe G. A. (2008). COI1 is a critical component of a receptor for jasmonate and the bacterial virulence factor coronatine. *Proc. Natl. Acad. Sci. U.S.A.* 105 7100–7105 18458331 10.1073/pnas.0802332105 PMC2383947

[B60] Kay S. Hahn S. Marois E. Hause G. Bonas U. (2007). A bacterial effector acts as a plant transcription factor and induces a cell size regulator. *Science* 318 648–651 17962565 10.1126/science.1144956

[B61] Kelley D. R. Estelle M. (2012). Ubiquitin-mediated control of plant hormone signaling. *Plant Physiol.* 160 47–55 22723083 10.1104/pp.112.200527 PMC3440220

[B62] Kitagawa M. Mukai H. Shibata H. Ono Y. (1995). Purification and characterization of a fatty acid-activated protein kinase (PKN) from rat testis. *Biochem. J. * 310(Pt 2) 657–664 7654208 10.1042/bj3100657 PMC1135946

[B63] Kloek A. P. Verbsky M. L. Sharma S. B. Schoelz J. E. Vogel J. Klessig D. F. (2001). Resistance to *Pseudomonas syringae* conferred by an *Arabidopsis thaliana* coronatine-insensitive (coi1) mutation occurs through two distinct mechanisms. *Plant J.* 26 509–522 11439137 10.1046/j.1365-313x.2001.01050.x

[B64] Kloppholz S. Kuhn H. Requena N. (2011). A secreted fungal effector of Glomus intraradices promotes symbiotic biotrophy. *Curr. Biol.* 21 1204–1209 21757354 10.1016/j.cub.2011.06.044

[B65] Laurie-Berry N. Joardar V. Street I. H. Kunkel B. N. (2006). The *Arabidopsis thaliana* JASMONATE INSENSITIVE 1 gene is required for suppression of salicylic acid-dependent defenses during infection by *Pseudomonas syringae*. *Mol. Plant Microbe Interact.* 19 789–800 16838791 10.1094/MPMI-19-0789

[B66] Lee S. Cheng H. King K. E. Wang W. He Y. Hussain A. (2002). Gibberellin regulates *Arabidopsis* seed germination via RGL2, a GAI/RGA-like gene whose expression is up-regulated following imbibition. *Genes Dev.* 16 646–658 11877383 10.1101/gad.969002 PMC155355

[B67] Li J. Brader G. Kariola T. Palva E. T. (2006). WRKY70 modulates the selection of signaling pathways in plant defense. *Plant J.* 46 477–491 16623907 10.1111/j.1365-313X.2006.02712.x

[B68] Li J. Brader G. Palva E. T. (2004). The WRKY70 transcription factor: a node of convergence for jasmonate-mediated and salicylate-mediated signals in plant defense. *Plant Cell* 16 319–331 14742872 10.1105/tpc.016980 PMC341906

[B69] Li L. Yu X. Thompson A. Guo M. Yoshida S. Asami T. (2009). *Arabidopsis* MYB30 is a direct target of BES1 and cooperates with BES1 to regulate brassinosteroid-induced gene expression. *Plant J.* 58 275–286 19170933 10.1111/j.1365-313X.2008.03778.x PMC2814797

[B70] Loake G. Grant M. (2007). Salicylic acid in plant defence–the players and protagonists. *Curr. Opin. Plant Biol.* 10 466–472 17904410 10.1016/j.pbi.2007.08.008

[B71] Lorenzo O. Chico J. M. Sanchez-Serrano J. J. Solano R. (2004). JASMONATE-INSENSITIVE1 encodes a MYC transcription factor essential to discriminate between different jasmonate-regulated defense responses in *Arabidopsis*. *Plant Cell* 16 1938–1950 15208388 10.1105/tpc.022319 PMC514172

[B72] Lorenzo O. Piqueras R. Sanchez-Serrano J. J. Solano R. (2003). ETHYLENE RESPONSE FACTOR1 integrates signals from ethylene and jasmonate pathways in plant defense. *Plant Cell* 15 165–178 12509529 10.1105/tpc.007468 PMC143489

[B73] Lorenzo O. Solano R. (2005). Molecular players regulating the jasmonate signalling network. *Curr. Opin. Plant Biol.* 8 532–540 16039901 10.1016/j.pbi.2005.07.003

[B74] Lumba S. Cutler S. Mccourt P. (2010). Plant nuclear hormone receptors: a role for small molecules in protein–protein interactions. *Annu. Rev. Cell Dev. Biol.* 26 445–469 20590451 10.1146/annurev-cellbio-100109-103956

[B75] Maor R. Jones A. Nuhse T. S. Studholme D. J. Peck S. C. Shirasu K. (2007). Multidimensional protein identification technology (MudPIT) analysis of ubiquitinated proteins in plants. *Mol. Cell. Proteomics* 6 601–610 17272265 10.1074/mcp.M600408-MCP200

[B76] McGrath K. C. Dombrecht B. Manners J. M. Schenk P. M. Edgar C. I. Maclean D. J. (2005). Repressor- and activator-type ethylene response factors functioning in jasmonate signaling and disease resistance identified via a genome-wide screen of *Arabidopsis* transcription factor gene expression. *Plant Physiol.* 139 949–959 16183832 10.1104/pp.105.068544 PMC1256008

[B77] Melotto M. Underwood W. He S. Y. (2008). Role of stomata in plant innate immunity and foliar bacterial diseases. *Annu. Rev. Phytopathol.* 46 101–122 18422426 10.1146/annurev.phyto.121107.104959 PMC2613263

[B78] Melotto M. Underwood W. Koczan J. Nomura K. He S. Y. (2006). Plant stomata function in innate immunity against bacterial invasion. *Cell* 126 969–980 16959575 10.1016/j.cell.2006.06.054

[B79] Meyer A. J. (2008). The integration of glutathione homeostasis and redox signaling. *J. Plant Physiol.* 165 1390–1403 18171593 10.1016/j.jplph.2007.10.015

[B80] Mittal S. Davis K. R. (1995). Role of the phytotoxin coronatine in the infection of *Arabidopsis thaliana* by *Pseudomonas syringae* pv. tomato. *Mol. Plant Microbe Interact.* 8 165–171 7539639 10.1094/mpmi-8-0165

[B81] Mosblech A. Thurow C. Gatz C. Feussner I. Heilmann I. (2011). Jasmonic acid perception by COI1 involves inositol polyphosphates in *Arabidopsis thaliana*. *Plant J.* 65 949–957 21205029 10.1111/j.1365-313X.2011.04480.x

[B82] Moscou M. J. Bogdanove A. J. (2009). A simple cipher governs DNA recognition by TAL effectors. *Science* 326 1501 10.1126/science.1178817 19933106

[B83] Navarro L. Bari R. Achard P. Lison P. Nemri A. Harberd N. P. (2008). DELLAs control plant immune responses by modulating the balance of jasmonic acid and salicylic acid signaling. *Curr. Biol.* 18 650–655 18450451 10.1016/j.cub.2008.03.060

[B84] Ndamukong I. Abdallat A. A. Thurow C. Fode B. Zander M. Weigel R. (2007). SA-inducible *Arabidopsis* glutaredoxin interacts with TGA factors and suppresses JA-responsive PDF1.2 transcription. *Plant J.* 50 128–139 17397508 10.1111/j.1365-313X.2007.03039.x

[B85] Niu Y. Figueroa P. Browse J. (2011). Characterization of JAZ-interacting bHLH transcription factors that regulate jasmonate responses in *Arabidopsis*. *J. Exp. Bot.* 62 2143–2154 21321051 10.1093/jxb/erq408 PMC3060693

[B86] Ohme-Takagi M. Shinshi H. (1995). Ethylene-inducible DNA binding proteins that interact with an ethylene-responsive element. *Plant Cell* 7 173–182 7756828 10.1105/tpc.7.2.173 PMC160773

[B87] Pauwels L. Barbero G. F. Geerinck J. Tilleman S. Grunewald W. Perez A. C. (2010). NINJA connects the co-repressor TOPLESS to jasmonate signalling. *Nature* 464 788–791 20360743 10.1038/nature08854 PMC2849182

[B88] Pauwels L. Goossens A. (2011). The JAZ proteins: a crucial interface in the jasmonate signaling cascade. *Plant Cell* 23 3089–3100 21963667 10.1105/tpc.111.089300 PMC3203442

[B89] Peng J. Carol P. Richards D. E. King K. E. Cowling R. J. Murphy G. P. (1997). The *Arabidopsis* GAI gene defines a signaling pathway that negatively regulates gibberellin responses. *Genes Dev.* 11 3194–3205 9389651 10.1101/gad.11.23.3194 PMC316750

[B90] Petersen M. Brodersen P. Naested H. Andreasson E. Lindhart U. Johansen B. (2000). *Arabidopsis* map kinase 4 negatively regulates systemic acquired resistance. *Cell* 103 1111–1120 11163186 10.1016/s0092-8674(00)00213-0

[B91] Pieterse C. M. Van Der Does D. Zamioudis C. Leon-Reyes A Van Wees S. C. (2012). Hormonal modulation of plant immunity. *Annu. Rev. Cell Dev. Biol.* 28 489–521 22559264 10.1146/annurev-cellbio-092910-154055

[B92] Plett J. M. Kemppainen M. Kale S. D. Kohler A. Legue V. Brun A. (2011). A secreted effector protein of *Laccaria bicolor* is required for symbiosis development. *Curr. Biol.* 21 1197–1203 21757352 10.1016/j.cub.2011.05.033

[B93] Potuschak T. Lechner E. Parmentier Y. Yanagisawa S. Grava S. Koncz C. (2003). EIN3-dependent regulation of plant ethylene hormone signaling by two *Arabidopsis* F box proteins: EBF1 and EBF2. *Cell* 115 679–689 14675533 10.1016/s0092-8674(03)00968-1

[B94] Qi T. Song S. Ren Q. Wu D. Huang H. Chen Y. (2011). The Jasmonate-ZIM-domain proteins interact with the WD-Repeat/bHLH/MYB complexes to regulate Jasmonate-mediated anthocyanin accumulation and trichome initiation in *Arabidopsis thaliana*. *Plant Cell* 23 1795–1814 21551388 10.1105/tpc.111.083261 PMC3123955

[B95] Robert-Seilaniantz A. Grant M. Jones J. D. (2011). Hormone crosstalk in plant disease and defense: more than just jasmonate-salicylate antagonism. *Annu. Rev. Phytopathol.* 49 317–343 21663438 10.1146/annurev-phyto-073009-114447

[B96] Santner A. Estelle M. (2009). Recent advances and emerging trends in plant hormone signalling. *Nature* 459 1071–1078 19553990 10.1038/nature08122

[B97] Saracco S. A. Hansson M. Scalf M. Walker J. M. Smith L. M. Vierstra R. D. (2009). Tandem affinity purification and mass spectrometric analysis of ubiquitylated proteins in *Arabidopsis*. *Plant J.* 59 344–358 19292762 10.1111/j.1365-313X.2009.03862.x PMC3639010

[B98] Schaller A. Stintzi A. (2009). Enzymes in jasmonate biosynthesis – structure, function, regulation. *Phytochemistry* 70 1532–1538 19703696 10.1016/j.phytochem.2009.07.032

[B99] Schornack S. Van Damme M. Bozkurt T. O. Cano L. M. Smoker M. Thines M. (2010). Ancient class of translocated oomycete effectors targets the host nucleus. *Proc. Natl. Acad. Sci. U.S.A.* 107 17421–17426 20847293 10.1073/pnas.1008491107 PMC2951462

[B100] Sheard L. B. Tan X. Mao H. Withers J. Ben-Nissan G. Hinds T. R. (2010). Jasmonate perception by inositol-phosphate-potentiated COI1-JAZ co-receptor. *Nature* 468 400–405 20927106 10.1038/nature09430 PMC2988090

[B101] Siewers V. Kokkelink L. Smedsgaard J. Tudzynski P. (2006). Identification of an abscisic acid gene cluster in the grey mold *Botrytis cinerea*. *Appl. Environ. Microbiol.* 72 4619–4626 16820452 10.1128/AEM.02919-05 PMC1489360

[B102] Silverstone A. L. Jung H. S. Dill A. Kawaide H. Kamiya Y. Sun T. P. (2001). Repressing a repressor: gibberellin-induced rapid reduction of the RGA protein in *Arabidopsis*. *Plant Cell* 13 1555–1566 11449051 10.1105/TPC.010047 PMC139546

[B103] Song S. Qi T. Huang H. Ren Q. Wu D. Chang C. (2011). The Jasmonate-ZIM domain proteins interact with the R2R3-MYB transcription factors MYB21 and MYB24 to affect Jasmonate-regulated stamen development in *Arabidopsis*. *Plant Cell* 23 1000–1013 21447791 10.1105/tpc.111.083089 PMC3082250

[B104] Spaepen S. Vanderleyden J. Remans R. (2007). Indole-3-acetic acid in microbial and microorganism-plant signaling. *FEMS Microbiol. Rev.* 31 425–448 17509086 10.1111/j.1574-6976.2007.00072.x

[B105] Splivallo R. Fischer U. Gobel C. Feussner I. Karlovsky P. (2009). Truffles regulate plant root morphogenesis via the production of auxin and ethylene. *Plant Physiol.* 150 2018–2029 19535471 10.1104/pp.109.141325 PMC2719122

[B106] Spoel S. H. Johnson J. S. Dong X. (2007). Regulation of tradeoffs between plant defenses against pathogens with different lifestyles. *Proc. Natl. Acad. Sci. U.S.A.* 104 18842–18847 17998535 10.1073/pnas.0708139104 PMC2141864

[B107] Spoel S. H. Koornneef A. Claessens S. M. Korzelius J. P. Van Pelt J. A. Mueller M. J. (2003). NPR1 modulates cross-talk between salicylate- and jasmonate-dependent defense pathways through a novel function in the cytosol. *Plant Cell* 15 760–770 12615947 10.1105/tpc.009159 PMC150028

[B108] Sun T. P. (2011). The molecular mechanism and evolution of the GA-GID1-DELLA signaling module in plants. *Curr. Biol.* 21 R338–R345 21549956 10.1016/j.cub.2011.02.036

[B109] Suzuki T. Miwa K. Ishikawa K. Yamada H. Aiba H. Mizuno T. (2001). The *Arabidopsis* sensor His-kinase, AHk4, can respond to cytokinins. *Plant Cell Physiol.* 42 107–113 11230563 10.1093/pcp/pce037

[B110] Tada Y. Spoel S. H. Pajerowska-Mukhtar K. Mou Z. Song J. Wang C. (2008). Plant immunity requires conformational changes [corrected] of NPR1 via *S*-nitrosylation and thioredoxins. *Science* 321 952–956 18635760 10.1126/science.1156970 PMC3833675

[B111] Thines B. Katsir L. Melotto M. Niu Y. Mandaokar A. Liu G. (2007). JAZ repressor proteins are targets of the SCF(COI1) complex during jasmonate signalling. *Nature* 448 661–665 17637677 10.1038/nature05960

[B112] Thomma B. P. Eggermont K. Penninckx I. A. Mauch-Mani B. Vogelsang R. Cammue B. P. (1998). Separate jasmonate-dependent and salicylate-dependent defense-response pathways in *Arabidopsis* are essential for resistance to distinct microbial pathogens. *Proc. Natl. Acad. Sci. U.S.A.* 95 15107–15111 9844023 10.1073/pnas.95.25.15107 PMC24583

[B113] Tyler L. Thomas S. G. Hu J. Dill A. Alonso J. M. Ecker J. R. (2004). Della proteins and gibberellin-regulated seed germination and floral development in *Arabidopsis*. *Plant Physiol.* 135 1008–1019 15173565 10.1104/pp.104.039578 PMC514135

[B114] Ueguchi C. Sato S. Kato T. Tabata S. (2001). The AHK4 gene involved in the cytokinin-signaling pathway as a direct receptor molecule in *Arabidopsis thaliana*. *Plant Cell Physiol.* 42 751–755 11479382 10.1093/pcp/pce094

[B115] Uppalapati S. R. Ishiga Y. Wangdi T. Kunkel B. N. Anand A. Mysore K. S. (2007). The phytotoxin coronatine contributes to pathogen fitness and is required for suppression of salicylic acid accumulation in tomato inoculated with *Pseudomonas syringae* pv. tomato DC3000. *Mol. Plant Microbe Interact*. 20 955–965 17722699 10.1094/MPMI-20-8-0955

[B116] Valls M. Genin S. Boucher C. (2006). Integrated regulation of the type III secretion system and other virulence determinants in *Ralstonia solanacearum*. *PLoS Pathog.* 2:e82 10.1371/journal.ppat.0020082 PMC1557829 16933989

[B117] Van der Does D. Leon-Reyes A. Koornneef A. Van Verk M. C. Rodenburg N. Pauwels L. (2013). Salicylic acid suppresses jasmonic acid signaling downstream of SCFCOI1-JAZ by targeting GCC promoter motifs via transcription factor ORA59. *Plant Cell* 10.1105/tpc.112.108548 [Epub ahead of print]. PMC3608790 23435661

[B118] van Loon L. C. Rep M. Pieterse C. M. (2006). Significance of inducible defense-related proteins in infected plants. *Annu. Rev. Phytopathol.* 44 135–162 16602946 10.1146/annurev.phyto.44.070505.143425

[B119] Vicente J. Cascon T. Vicedo B. Garcia-Agustin P. Hamberg M. Castresana C. (2012). Role of 9-lipoxygenase and alpha-dioxygenase oxylipin pathways as modulators of local and systemic defense. *Mol. Plant* 5 914–928 22199234 10.1093/mp/ssr105

[B120] Vlot A. C. Dempsey D. A. Klessig D. F. (2009). Salicylic acid, a multifaceted hormone to combat disease. *Annu. Rev. Phytopathol.* 47 177–206 19400653 10.1146/annurev.phyto.050908.135202

[B121] Wang K. L. Li H. Ecker J. R. (2002). Ethylene biosynthesis and signaling networks. *Plant Cell* 14(Suppl.) S131–S151 12045274 10.1105/tpc.001768 PMC151252

[B122] Wang W. Esch J. J. Shiu S. H. Agula H. Binder B. M. Chang C. (2006). Identification of important regions for ethylene binding and signaling in the transmembrane domain of the ETR1 ethylene receptor of *Arabidopsis*. *Plant Cell* 18 3429–3442 17189345 10.1105/tpc.106.044537 PMC1785413

[B123] Wasternack C. (2007). Jasmonates: an update on biosynthesis, signal transduction and action in plant stress response, growth and development. *Ann. Bot.* 100 681–697 17513307 10.1093/aob/mcm079 PMC2749622

[B124] Weingart H. Ullrich H. Geider K. Volksch B. (2001). The role of ethylene production in virulence of *Pseudomonas syringae* pvs. *glycinea* and *phaseolicola*. *Phytopathology* 91 511–518 18943596 10.1094/PHYTO.2001.91.5.511

[B125] Weingart H. Volksch B. (1997). Ethylene production by *Pseudomonas syringae* Pathovars in vitro and in planta. *Appl. Environ. Microbiol.* 63 156–161 16535480 10.1128/aem.63.1.156-161.1997 PMC1389095

[B126] Wild M. Daviere J. M. Cheminant S. Regnault T. Baumberger N. Heintz D. (2012). The *Arabidopsis* DELLA RGA-LIKE3 is a direct target of MYC2 and modulates jasmonate signaling responses. *Plant Cell* 24 3307–3319 22892320 10.1105/tpc.112.101428 PMC3462633

[B127] Withers J. Yao J. Mecey C. Howe G. A. Melotto M. He S. Y. (2012). Transcription factor-dependent nuclear localization of a transcriptional repressor in jasmonate hormone signaling. *Proc. Natl. Acad. Sci. U.S.A.* 109 20148–20153 23169619 10.1073/pnas.1210054109 PMC3523844

[B128] Wu Y. Zhang D. Chu J. Y. Boyle P. Wang Y. Brindle I. D. (2012). The *Arabidopsis* NPR1 protein is a receptor for the plant defense hormone salicylic acid. *Cell Rep.* 1 639–647 22813739 10.1016/j.celrep.2012.05.008

[B129] Xie D. X. Feys B. F. James S. Nieto-Rostro M. Turner J. G. (1998). COI1: an *Arabidopsis* gene required for jasmonate-regulated defense and fertility. *Science* 280 1091–1094 9582125 10.1126/science.280.5366.1091

[B130] Yamada H. Suzuki T. Terada K. Takei K. Ishikawa K. Miwa K. (2001). The *Arabidopsis* AHK4 histidine kinase is a cytokinin-binding receptor that transduces cytokinin signals across the membrane. *Plant Cell Physiol.* 42 1017–1023 11577198 10.1093/pcp/pce127

[B131] Yang D. L. Yao J. Mei C. S. Tong X. H. Zeng L. J. Li Q. (2012). Plant hormone jasmonate prioritizes defense over growth by interfering with gibberellin signaling cascade. *Proc. Natl. Acad. Sci. U.S.A.* 109 E1192–E1200 22529386 10.1073/pnas.1201616109 PMC3358897

[B132] Zamioudis C. Pieterse C. M. (2012). Modulation of host immunity by beneficial microbes. *Mol. Plant Microbe Interact.* 25 139–150 21995763 10.1094/MPMI-06-11-0179

[B133] Zander M. Chen S. Imkampe J. Thurow C. Gatz C. (2012). Repression of the *Arabidopsis* thaliana jasmonic acid/ethylene-induced defense pathway by TGA-interacting glutaredoxins depends on their C-terminal ALWL motif. *Mol. Plant* 5 831–840 22207719 10.1093/mp/ssr113

[B134] Zander M. La Camera S. Lamotte O. Metraux J. P. Gatz C. (2009). *Arabidopsis thaliana* class-II TGA transcription factors are essential activators of jasmonic acid/ethylene-induced defense responses. *Plant J.* 61 200–210 19832945 10.1111/j.1365-313X.2009.04044.x

[B135] Zhu Z. An F. Feng Y. Li P. Xue L. A M. (2011). Derepression of ethylene-stabilized transcription factors (EIN3/EIL1) mediates jasmonate and ethylene signaling synergy in *Arabidopsis*. *Proc. Natl. Acad. Sci. U.S.A.* 108 12539–12544 21737749 10.1073/pnas.1103959108 PMC3145709

